# Humanin: A Novel Central Regulator of Peripheral Insulin Action

**DOI:** 10.1371/journal.pone.0006334

**Published:** 2009-07-22

**Authors:** Radhika H. Muzumdar, Derek M. Huffman, Gil Atzmon, Christoph Buettner, Laura J. Cobb, Sigal Fishman, Temuri Budagov, Lingguang Cui, Francine H. Einstein, Aruna Poduval, David Hwang, Nir Barzilai, Pinchas Cohen

**Affiliations:** 1 Department of Pediatrics, Children's Hospital at Montefiore, Institute for Aging Research, Diabetes Research and Training Center, Albert Einstein College of Medicine, Bronx, New York, United States of America; 2 Department of Medicine, Albert Einstein College of Medicine, Bronx, New York, United States of America; 3 Department of Medicine, Mount Sinai School of Medicine, New York, New York, United States of America; 4 Department of Obstetrics & Gynecology and Women's Health, Albert Einstein College of Medicine, Bronx, New York, United States of America; 5 Department of Pediatrics, Mattel Children's Hospital, Los Angeles, California, United States of America; Mayo Clinic College of Medicine, United States of America

## Abstract

**Background:**

Decline in insulin action is a metabolic feature of aging and is involved in the development of age-related diseases including Type 2 Diabetes Mellitus (T2DM) and Alzheimer's disease (AD). A novel mitochondria-associated peptide, Humanin (HN), has a neuroprotective role against AD-related neurotoxicity. Considering the association between insulin resistance and AD, we investigated if HN influences insulin sensitivity.

**Methods and Findings:**

Using state of the art clamp technology, we examined the role of central and peripheral HN on insulin action. Continuous infusion of HN intra-cerebro-ventricularly significantly improved overall insulin sensitivity. The central effects of HN on insulin action were associated with activation of hypothalamic STAT-3 signaling; effects that were negated by co-inhibition of hypothalamic STAT-3. Peripheral intravenous infusions of novel and potent HN derivatives reproduced the insulin-sensitizing effects of central HN. Inhibition of hypothalamic STAT-3 completely negated the effects of IV HN analog on liver, suggesting that the hepatic actions of HN are centrally mediated. This is consistent with the lack of a direct effect of HN on primary hepatocytes. Furthermore, single treatment with a highly-potent HN analog significantly lowered blood glucose in Zucker diabetic fatty rats. Based upon the link of HN with two age-related diseases, we examined if there were age associated changes in HN levels. Indeed, the amount of detectable HN in hypothalamus, skeletal muscle, and cortex was decreased with age in rodents, and circulating levels of HN were decreased with age in humans and mice.

**Conclusions:**

We conclude that the decline in HN with age could play a role in the pathogenesis of age-related diseases including AD and T2DM. HN represents a novel link between T2DM and neurodegeneration and along with its analogues offers a potential therapeutic tool to improve insulin action and treat T2DM.

## Introduction

Humanin (HN) is a 24 amino acid polypeptide (Met- Ala- Pro- Arg- Gly- Phe- Ser- Cys- Leu- Leu- Leu- Leu- Thr- Ser- Glu- Ile- Asp- Leu- Pro- Val- Lys- Arg- Arg- Ala; M.W. = 2656.3 Da) that was first identified from a cDNA library from the surviving neurons of human Alzheimer's disease (AD) brain [1]. Since its initial discovery, several cDNAs sharing sequence homology to HN have been identified in plants, nematodes, and rodents demonstrating that HN is evolutionarily conserved [2]. HN is transcribed from an open reading frame within the mitochondrial 16S ribosomal RNA. Endogenous HN is both an intracellular and secreted protein and has been detected in normal mouse testis and colon at specific stages of development [3]. In addition to brain, colon and testis, we have shown the presence of HN by western blot in rodent heart, ovary, pancreas and kidney (unpublished data). In addition, our group has demonstrated the presence of HN in cerebral spinal fluid (CSF), seminal fluid and plasma, with levels in the biologically active range (Cohen & Hwang, unpublished data).

Despite little being known regarding the regulation of HN production *in vivo*, the major role of HN is believed to be promoting cell survival. Indeed, HN has a well-described role in neuroprotection against cell death associated with AD [1], from AD-specific insults [4], prion induced apoptosis [5] and chemically-induced neuronal damage [6]. Interestingly, a highly-potent analogue of HN, termed HNG, (HN in which the serine at position 14 is replaced by glycine), reverses the learning and memory impairment induced by scopolamine in mice [6] and also has rescue activity against memory impairment caused by AD-related insults *in vivo* [7]. The protection of HN from AD-related cytotoxicity has also been demonstrated in non-neuronal cells such as cerebrovascular smooth muscle [8], rat phaeochromocytoma cells and lymphocytes under serum-deprived conditions [9].

The anti-apoptotic potential of HN appears to be dependent upon the formation of homodimers, as interfering with this process completely blocks its ability to suppress cell death [10]. Once dimerized, HN directly interacts with a variety of pro-apoptotic proteins, including Bax-related proteins [2] and insulin-like growth factor binding protein-3 (IGFBP-3) [11]. It has been shown that HN protects against apoptosis by binding pro-apoptotic Bax, inhibiting its mitochondrial localization, and attenuating Bax-mediated apoptosis activation [2]. HN was also shown to act directly on Bax in isolated mitochondria suggesting that a cell surface receptor may not be required for its anti-apoptotic action [2] though some studies suggest involvement of the cell surface receptor FPRL-1 [12], [13]. A recent study has demonstrated that HN protects neurons by binding to a complex or complexes involving CNTFR/WSX-1/gp130 [13]. Moreover, neuronal protection by HN involves activation of tyrosine kinases and STAT-3 phosphorylation [14], while the inhibition of tyrosine kinases or the use of dominant negative STAT3 prevented the anti-apoptotic action of HN. Furthermore, HN delays apoptosis in K562 cells by downregulation of p38 MAP kinase [15] and prevents cell death in a constitutively activated Jun N-terminal kinase (JNK) cell line, suggesting that an important mechanism of cell protection could be via interfering with JNK activity [16].

The interaction between HN and IGFBP-3 is especially interesting since IGFBP-3 and HN have opposing roles on cell survival, such that HN protects against while IGFBP-3 induces cell death [17]. We previously demonstrated that HN physically binds with IGFBP-3 and that this interaction prevents the activation of caspases [11]. More recently, we showed that IGFBP-3, independent of IGF-1, induces insulin resistance both at the liver and periphery through the hypothalamus as well as by direct action [18], [19]. Based upon the molecular interaction between HN and IGFBP-3 and the emerging link between AD and insulin resistance [20], we hypothesized that HN, in addition to its neuroprotective roles, may serve as a centrally-acting regulator of glucose homeostasis. In a series of experiments, we examined the role of HN in glucose metabolism and its potential mechanism of action, including its interaction with IGFBP-3, by using HN and a variety of HN analogs. We also examined if there were changes in the levels of HN in circulation and in tissues with age.

## Methods

### Animal preparation for in vivo experiments

**Figure 1:**
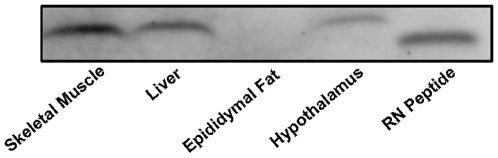
Protein level of the rat homolog to HN, termed rattin (RN), in metabolically-relevant tissues in male S–D rats. A total of 30 µg of rat skeletal muscle (lane 1), liver (lane 2), epididymal fat (lane 3) and hypothalamic protein (lane 4) and 1.5 ng of synthetic RN peptide (lane 5) were loaded. RN protein was detected in muscle, liver and hypothalamus but not in epididymal fat (lane 3).

We studied 92 three-month-old male Sprague−Dawley rats (S–D, Charles River Laboratories) and 9 three-month old Zucker Diabetic Fatty (ZDF) rats (Harlan). Rats were housed in individual cages and subjected to a standard light−dark cycle (12L∶12D). Depending on the study group the rats received ICV and IV catheters. For ICV studies, S–D rats were prepared for clamps as described in Fig. 1A. Three weeks before the *in vivo* studies, catheters were implanted in the third cerebral ventricle [21]. One week before clamp experiments, catheters were placed in the right internal jugular vein and left carotid artery [22]. ZDF rats were allowed to acclimate for 1 wk and glucose levels were monitored for the appearance of diabetes (>200 mg/dL). All animals became diabetic within 2 weeks of arrival. Once diabetic, animals had indwelling catheters inserted into the right internal jugular vein and the left carotid artery under anesthesia. Recovery was monitored until body weight was within 3% of the pre-operative weight (∼5–6 days). The study protocol was reviewed and approved by the Institutional Animal Care and Use Committee of the Albert Einstein College of Medicine.

### In vivo Intra-cerebro ventricular and Intravenous studies, analysis of glucose fluxes and hormone measurements

*Intra-cerebro ventricular (ICV) studies*: Food was removed for ∼5 h before the *in vivo* protocol. All studies lasted 360 min and included a 120-min equilibration period, a 120-min basal period for assessment of the basal glucose turnover, and a 120-min pancreatic or hyperinsulinemic clamp period. A primed-continuous ICV infusion of HN, F6AHN (total dose of 20 ug), dimerization deficient (DD) HN, STAT-3 inhibitor (75 pmol) , or artificial cerebro-spinal fluid (aCSF) was initiated at t = 0 and maintained throughout the experiment (Fig. 1A) by an infusion pump. Pancreatic insulin-clamp studies (1 mU/kg/min), approximating the basal fasting state with circulating insulin levels around 1.4 ng/ml, or euglycemic-hyperinsulinemic clamps (3 mU/kg/min), corresponding to the post-prandial state with circulating insulin levels around 4.5 ng/ml, were performed in conscious, unrestrained, catheterized rats for the last 120 min as described previously [19], [22].

In the intravenous (IV) studies, HN (0.375 mg/kg/hr), IGFBP-3 (0.06 mg/kg/hr), or analogs of HN such as F6AHN (0.375 mg/kg/hr) and HNGF6A (0.05 mg/kg/hr) were infused intravenously and hyperinsulinemic clamps were performed as described previously [18]. The HN analog F6AHN has a single amino acid substitution of alanine for phenylalanine in position 6; this substitution abrogates IGFBP-3 binding. Another HN analog HNG has an amino acid substitution at position 14 (glycine for serine) that increases the biologic potency by ∼1000 fold. A third HN analog, HNGF6A, contains both of these substitutions at positions 6 and 14, resulting in a potent non-IGFBP-3 binding peptide. DD HN analog has an amino acid substitution of alanine with serine at position 7 resulting in a peptide that fails to dimerize. A subgroup of animals that received IV HNGF6A also received ICV STAT-3 inhibitor [23].

The protocol followed during the insulin clamp study was similar to that previously described. Briefly, a primed-continuous infusion of regular insulin ( 1 or 3 milliunits/kg·min) was administered, and a variable infusion of a 25% dextrose solution was started and periodically adjusted to clamp the plasma glucose concentration at 7–8 mM. Somatostatin (1.5 µg/kg·min) was also infused in all the groups to prevent endogenous insulin secretion and to control for possible effects of the ICV infusions on the endocrine pancreas. Estimation of glucose, glucose fluxes, insulin and FFA were done as described previously [19], [22]. HN measurements were performed by an in-house ELISA utilizing an affinity purified HN antibody, which has a detection limit of 0.1 ng/ml (Hwang and Cohen, Personal communication).

Chronically catheterized ZDF rats were studied while awake, unrestrained and unstressed. Early in the morning, ZDF rats had food removed from their hoppers and animals were prepared for the procedure. Initially, rats were infused with a 100 ug HNGF6A bolus (0.5 ug/uL) (*n* = 5) or 200 uL saline (*n* = 4) into the carotid artery. Glucose levels were then monitored for 4 hours by sampling venous blood and glucose levels were determined in whole blood using glucose strips (One Touch Ultra, LifeScan Inc., Milpitas, CA).

### Signaling studies and Protein analysis

For acute signaling studies, rats were injected ICV with either aCSF, 20 ug of HN or F6AHN (dissolved in aCSF) over 5 min, and sacrificed 30 min later. At sacrifice, liver, skeletal muscle (quadriceps) and mediobasal hypothalamus (MBH) were rapidly excised and snap frozen in liquid nitrogen. The MBH was identified caudally by the mammillary bodies, rostrally by the optic chiasm, laterally by the optic tract, and superiorly by the apex of the hypothalamic third ventricle. Protein was then extracted from tissues and western blot analysis was performed as described previously [23]. Briefly, following incubation with the appropriate antibody, membranes were scanned using the LI-COR Odyssey (LI-COR, Lincoln, NE, USA) and quantified using Odyssey 2.0 software based on direct fluorescence measurement. Antibodies to STAT-3, pSTAT-3^ Tyr705^, AKT, pAKT^S473^, ACC, GAPDH, Insulin receptor (IR) and β-Tubulin were all obtained from Cell Signaling (Danvers, MA). The Dot blots were performed by blotting 50 ng of HN, HN-analogues, and IGF on nitrocellulose, incubating with 125-I-labeled-IGFBP-3, autoradiography and scanning. RN protein levels in tissues were measured using a rabbit polyclonal antibody to RN (1∶500, Sigma, St. Louis, MO) utilizing standard Western blotting techniques and imaged by chemiluminesence using a FujiFilm LAS-3000 bioimager (Valhalla, NY).

### Isolation of primary hepatocytes, hepatic glucose production and signaling pathways

Single-cell suspensions of hepatocytes were obtained from perfusions of Sprague–Dawley rats using the procedure of Berry [24] and the perfusion mixture of Leffert et al [25]. Hepatocyte glucose production was estimated after a 24 hr treatment with HN, F6AHN or saline as described before [26]. In a separate set of experiments, hepatocytes were lysed, and protein extracted in RIPA lysis buffer at 30, 60 and 360 min post treatment with HN, F6AHN and saline. The cell lysates were then subject to western blotting for evidence of STAT-3 and AKT activation.

### Statistical Analyses

All values shown are expressed as means±SE. When comparing two groups for analysis such as protein densitometry, independent two-tailed t test was used. For variables such as HGP or Rd involving more than 2 groups, one way ANOVA was used. When pre vs. post clamp comparisons for variables such as glycolysis and glycogen synthesis were made, two- way ANOVA (group×time) was used. For time course measures such as GIR, ANOVA with repeated measures on time was used. For each statistically significant *F* value observed for the main effect or interaction, a two-tailed post hoc test (Tukey's) was applied to determine individual differences between means. Differences were considered to be statistically significant when *P*≤0.05.

## Results

### HN is present in metabolically relevant tissues

To confirm the presence of rattin (RN), the rat homolog to HN, we analyzed the expression level of RN protein in metabolically relevant tissues including the skeletal muscle, liver, fat and hypothalamus. As demonstrated by western blot (Fig. 1), RN is expressed in muscle, liver and hypothalamus but was not detected in epididymal fat. The observed band for RN in the hypothalamus runs slightly higher than in other tissues. This is consistent with the observation by Tajima et al. [3] who showed that HN derived from lipid-enriched tissues in rodents produces a HN band that is shifted slightly higher (2–3 kD) than other tissues and that lipid extraction resulted in downward migration of the HN band. Furthermore, similar to HN in mouse tissues, endogenous RN runs at a slightly higher molecular weight than synthetic human peptide due to the presence of a C-terminal tail on endogenous RN.

### ICV HN increases systemic insulin sensitivity

**Figure 2:**
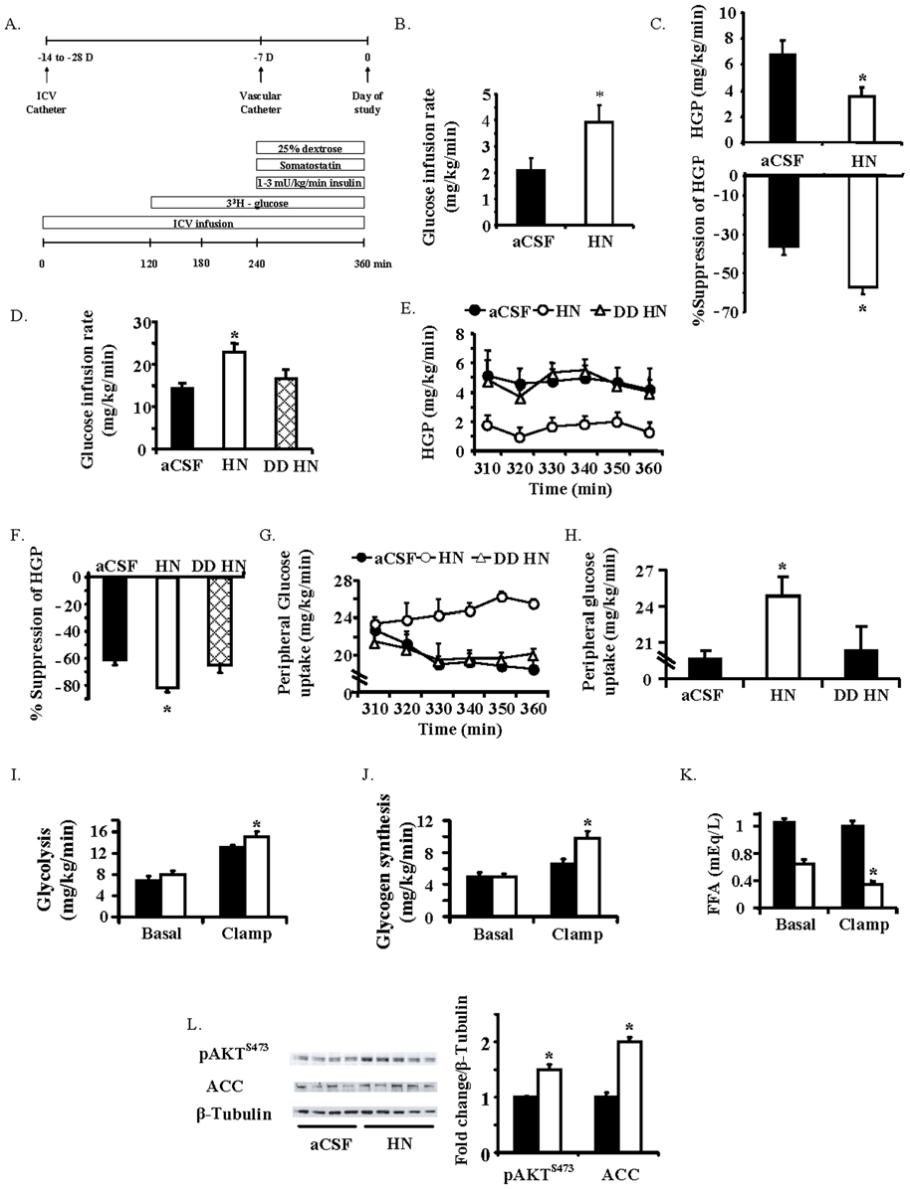
ICV HN increases peripheral insulin sensitivity. A) Schematic representation of the experimental design for the ICV studies; the upper panel demonstrates the time line for the surgical procedures and the lower panel demonstrates the protocol on the day of the clamp. B) Glucose infusion rate (GIR) and C) Hepatic glucose production (HGP) and degree of suppression of HGP by artificial cerebrospinal fluid (aCSF) and 0.16 µg/kg/min HN infusions (*n* = 5 each) during a basal pancreatic clamp. D) GIR, E) HGP and F) degree of HGP suppression during a hyperinsulinemic clamp with ICV infusion of aCSF, HN and a DDHN peptide (*n* = 7 each). G) Time course and H) last hour average of effects of ICV aCSF, HN and DDHN on peripheral glucose uptake during a hyperinsulinemic clamp. Effect of aCSF (black bars) and HN (white bars) on I) glycolysis, J) glycogen synthesis, and K) suppression of free fatty acids (FFA) in response to insulin. L) pAKT and acetyl CoA carboxylase (ACC) in skeletal muscle during insulin clamp in response to ICV aCSF and HN (*n* = 7 each). Values are means±SE. *Significantly different from other experimental groups, *P*<0.05.

Under basal insulin levels during pancreatic-euglycemic clamp (1.42±0.08 vs. 1.41±0.03 ng/ml in controls vs. HN, N.S), a higher glucose infusion rate (GIR) was required to maintain euglycemia in the groups that received ICV HN (Fig. 2B). Glucose kinetics using tracer methodology demonstrated that the need for a higher rate of glucose infusion (Fig. 2B) was due to enhanced hepatic insulin sensitivity, leading to a significant decrease in hepatic glucose production (HGP) (*P*<0.05, Fig. 2C).

Under physiologic hyperinsulinemic clamp conditions (insulin levels of 3.9±0.2 vs. 4.6±0.6 ng/ml in controls vs. HN, N.S), GIR was significantly higher in the ICV HN group (*P*<0.05, Fig. 2D). This effect was secondary to suppression of HGP (*P*<0.05, Fig. 2E, F) and enhanced skeletal muscle glucose uptake (*P*<0.05, Fig. 2G, H), features of enhanced hepatic and peripheral insulin sensitivity. HN was undetectable in the circulation during ICV infusion confirming that there was no leak of ICV infused HN (data not shown). The degree of suppression of HGP, an indicator of hepatic insulin sensitivity, was significantly higher in HN infused groups (12.1±0.9 to 4.7±0.4 in controls vs. 12.8±0.7 to 2.3±0.4 in HN infused groups, *P*<0.05). The dramatic increase in GIR with central HN is not only due to effects on HGP, but also through an increase in peripheral glucose uptake (*P*<0.05, Fig. 2G–H). Using tracer methodology, we demonstrate that the HN-induced increase in glucose uptake is associated with increases in both glycolysis and glycogen synthesis (*P*<0.05, Figs. 2I & 2J). Furthermore, insulin-induced suppression of free fatty acid (FFA) levels was significantly enhanced with ICV HN (Fig. 2K), reflecting an overall improvement in peripheral insulin sensitivity. In addition, we assessed the effects of DD HN on glucose metabolism since dimerization is an essential step in the cytoprotective effects of HN [27]. The infusion of DD HN ICV had no effect on overall glucose metabolism (Fig. 2D–H). Specifically, there were no differences in GIR (Fig. 2D), HGP (Fig. 2E, F) or peripheral glucose uptake (Fig. 2G, H) between the groups that received the DD HN and controls.

### Central HN activates mediators of insulin action in skeletal muscle

Consistent with enhanced glucose uptake, insulin signaling in the skeletal muscle was significantly increased during ICV HN infusion, in spite of similar circulating insulin levels (Fig. 2L). We observed increased phosphorylation of the insulin sensitive AKT (pAKT^S473^) and Acetyl-CoA Carboxylase (pACC^Ser79^) in skeletal muscle with HN. We also studied potential direct effects of HN on other signaling pathways; however, no significant differences in liver pAKT^S473^, PGC-1b, Fatty Acid Synthase (FAS), p38 MAPK, CREB or ACC were demonstrable between the HN and control groups (data not shown).

### Activation of STAT-3 in the hypothalamus is critical for the effects of HN on glucose metabolism

**Figure 3:**
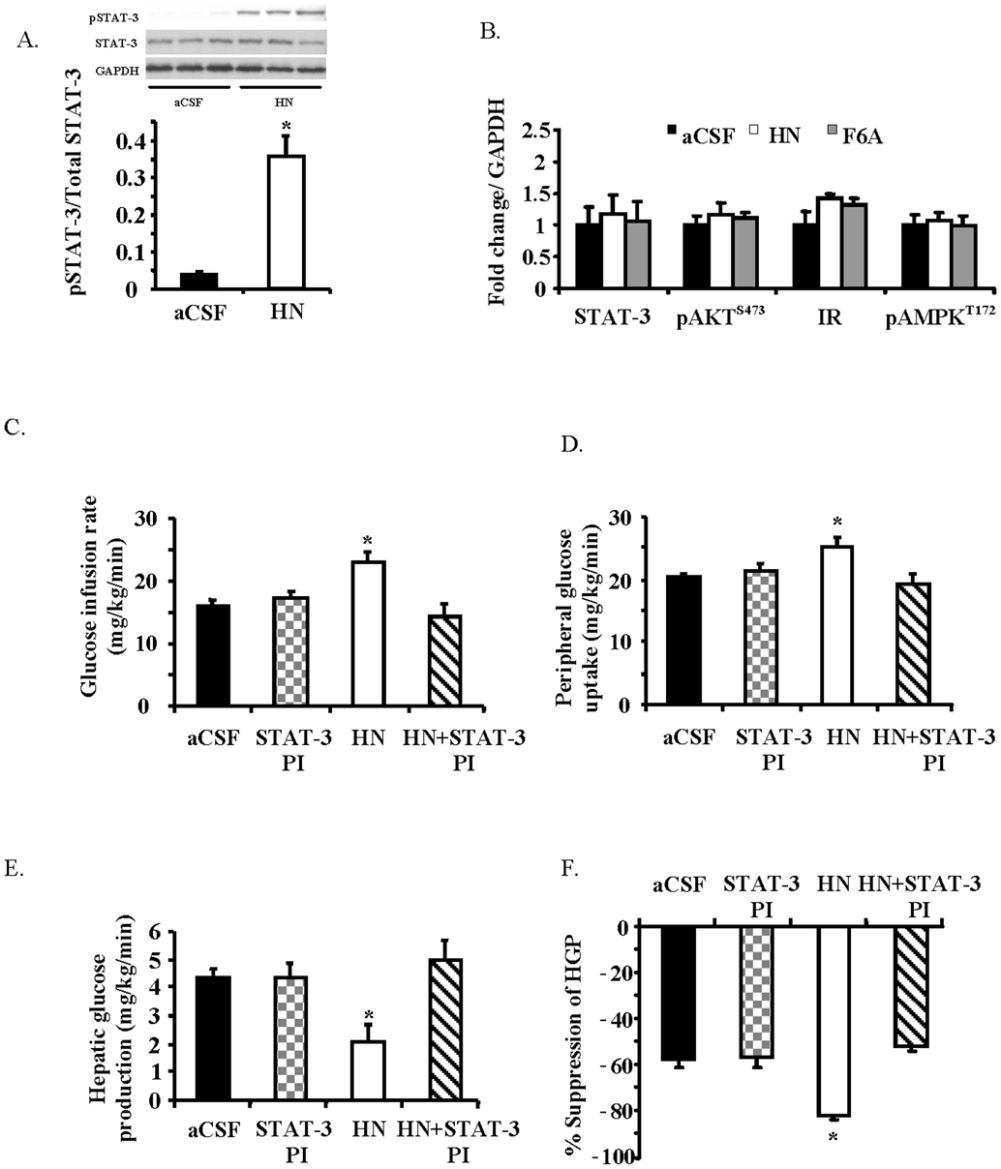
Central effects of HN on glucose metabolism involve hypothalamic STAT-3 activation. A) Effects of ICV HN or aCSF on pSTAT-3^Tyr705^ levels in hypothalamic protein extracts. B) Effects of ICV HN and F6AHN on the levels of totalSTAT-3, pAKT^S473^, insulin receptor (IR) and pAMPK^Thr172^ in the hypothalamus (aCSF -black bars, HN -white bars, F6AHN-grey, *n* = 5 each). Effects of a STAT-3 inhibitor co-infused ICV with HN or aCSF (*n* = 6 each) on C) GIR, D) peripheral glucose uptake, and E) HGP and degree of suppression of HGP. Values are means±SE. *Significantly different from other experimental groups, *P*<0.05.

Since HN has been shown to work via STAT-3 activation, we examined the effect of acute HN ICV infusion on STAT-3 phosphorylation (pSTAT-3^Tyr705^) in the hypothalamus. As presented in Fig. 3A, HN infusion resulted in a 10-fold increase in STAT-3 phosphorylation in hypothalamic tissues within an hour of ICV HN infusion (*P*<0.05). In contrast, no effect was observed on phosphorylation levels in other signaling kinases in the hypothalamus including pAKT^S473^ (Fig. 3B). To test the role of STAT-3 activation in the mediation of HN actions, we co-infused HN along with a STAT-3 inhibitor [28] ICV and showed that the effects of HN are completely attenuated in the presence of the STAT-3 inhibitor (Fig. 3C–3F). The hypothalamic delivery of the STAT-3 inhibitor showed no effect on its own, yet completely abolished HN-induced suppression of HGP and peripheral glucose uptake.

### Hypothalamic IGFBP-3 tempers the effects of HN on hepatic insulin action

**Figure 4:**
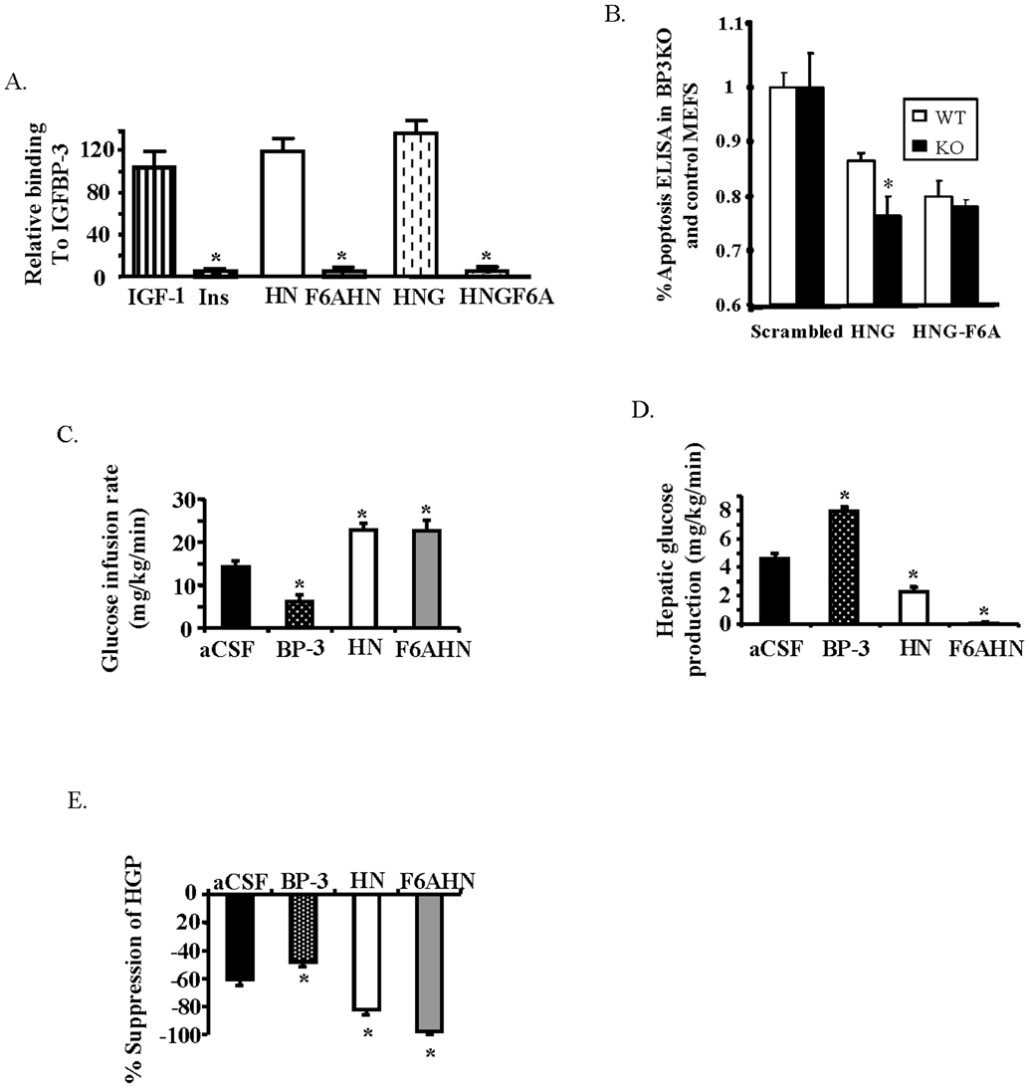
IGFBP-3 modulates the effects of HN on insulin sensitivity. A) IGFBP-3 binding of HN and HN analogues measured by densitometry of dot blots probed with radiolabeled IGFBP-3 (Ins = Insulin). B) Mouse embryonic fibroblasts generated from wild-type or IGFBP-3 knockout mice were incubated in serum-free media for 24 h followed by incubation with 100 nM HNG or HNGF6A for 24 h. Apoptosis was assessed by ELISA for fragmentation of histone-associated DNA. (*n* = 4). Effects of ICV IGFBP-3, HN or F6AHN during a hyperinsulinemic clamp (*n* = 6 each)on C) GIR, D) HGP and E) degree of suppression of HGP. *Significantly different from other experimental groups, *P*<0.05.

We have previously shown that IGFBP-3 is present in the hypothalamus and inhibits hepatic insulin action via a hypothalamic mechanism [19]. We have separately shown that IGFBP-3 also binds HN and antagonizes its survival effects [11]. To assess if IGFBP-3 attenuates the effects of HN on insulin action, we utilized a series of HN analogues that do not bind to IGFBP-3 (Fig. 4A). Non-IGFBP-3 binding homologues are more potently active as a direct result of the loss of IGFBP-3 binding, as exemplified in apoptosis studies using mouse embryo fibroblasts from IGFBP-3 knockout mice (Fig. 4B). We hypothesized that if IGFBP-3 inhibits the effects of HN on glucose metabolism, non-IGFBP-3 binding analogues of HN would have a greater effect. As expected, infusion of IGFBP-3 ICV led to a decrease in GIR, but both ICV HN and the non-BP3-binding HN analogue (F6AHN) increased GIR (*P*<0.05, Fig. 4C). Remarkably, under similar insulin levels, (3.9±0.2, 4.6±0.6 and 5.0±1.0 ng/ml in controls, HN, and F6AHN respectively, N.S) F6AHN enhanced hepatic insulin action to an even greater extent than HN. This effect was primarily due to a near-complete inhibition of HGP (from 12.1±0.9 to 4.7±0.4 in controls vs. 11.0±0.3 to 0.6±1.0 in F6AHN , *P*<0.05, Fig. 4D–E), effects that cannot be elicited in rodents even with pharmacological levels of insulin.

### Peripheral administration of a potent HN analog improves insulin sensitivity

**Figure 5:**
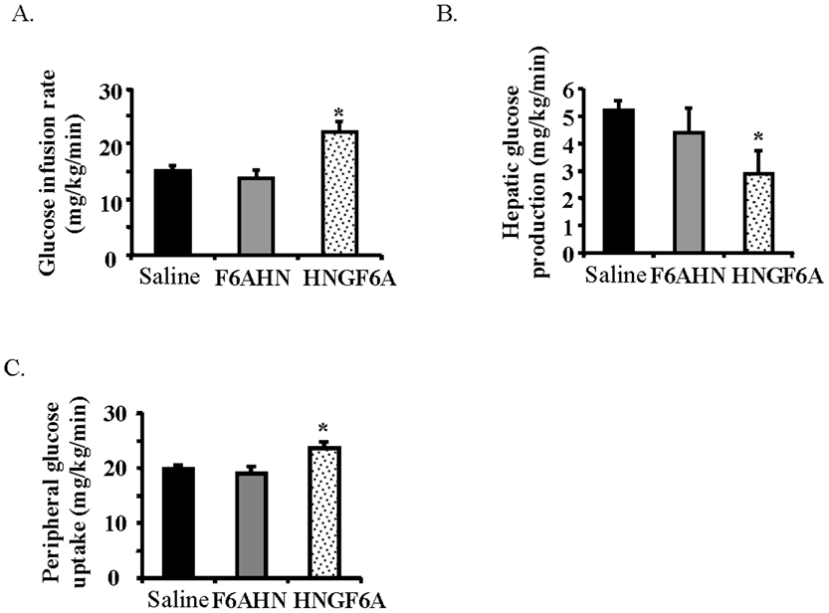
Effects of peripheral administration of the potent HN analog, HNGF6A, on glucose metabolism. Effect of IV saline, F6AHN and HNGF6A (*n* = 6 each) on A) GIR, B) HGP, and C) glucose uptake. Values are means±SE. *Significantly different from other experimental groups, *P*<0.05.

We then assessed if peripheral administration of HN can reproduce the central effects of HN. We infused HN or F6AHN intravenously (at a rate of 0.375 mg/kg/hr) during a hyperinsulinemic clamp to examine the effect of these compounds on glucose fluxes. HN and F6AHN, though potent when given centrally, did not significantly alter insulin action when infused intravenously at the given dose (data not shown). We then engineered a more potent non-IGFBP-3 binding analogue of HN, with two substitutions, S14G (HNG, an analog shown to be more potent), along with F6A (resulting in non-IGFBP3 binding) resulting in a more potent, non-IGFBP-3-binding- analogue of HN, termed HNGF6A. When HNGF6A was infused intravenously (0.05 mg/kg/hr) during a hyperinsulinemic clamp (insulin levels of 4.5±0.3 vs. 4.1±0.2 ng/ml in controls vs. HNGF6A, N.S), glucose infusion rates during clamp were significantly higher (*P*<0.05, Fig. 5A) and this was due to an enhancement of peripheral glucose uptake and suppression of HGP (*P*<0.05, Fig. 5B, Fig. 5C). Thus, intravenous HNGF6A, a more potent analog of HN, is able to reproduce the potent effects of central HN on insulin sensitivity.

### Effects of the potent HN analog, HNGF6A on hepatic insulin sensitivity are mediated through the hypothalamus

**Figure 6:**
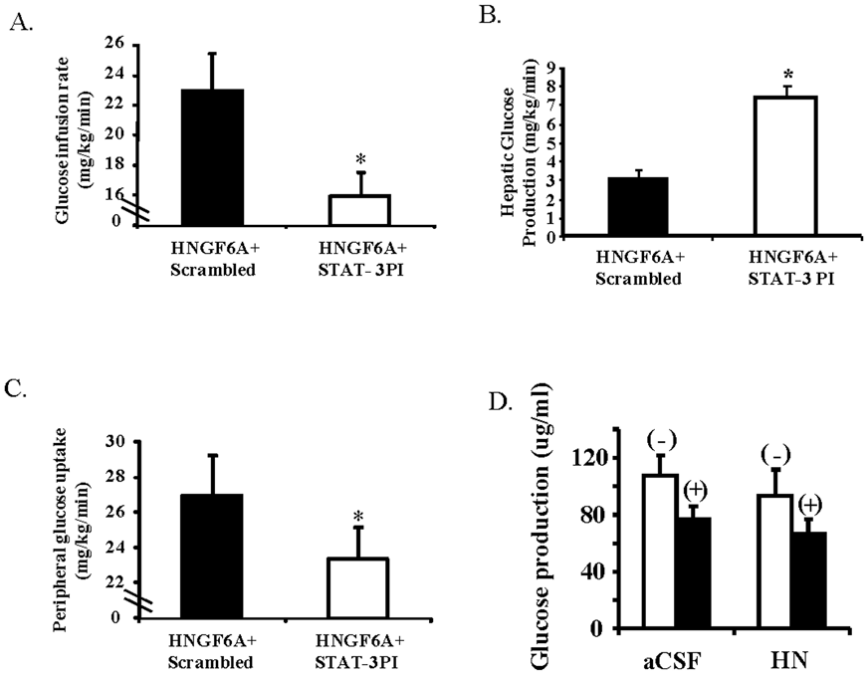
Effects of IV HNGF6A in the presence of hypothalamic STAT-3 inhibitor on A) GIR, B) HGP and C) peripheral glucose uptake. D) Effect of HN on glucose production from primary isolated hepatocytes treated with (+), or without (−) insulin. Values are means±SE. *Significantly different from other experimental groups, *P*<0.05.

To examine the role of the hypothalamus in mediating the effects of peripherally administered HN analogue, we infused HNGF6A peripherally in the presence of hypothalamic inhibition of STAT-3 and studied glucose fluxes under insulin clamps. The insulin levels achieved were similar and glucose was clamped at basal levels. In the presence of hypothalamic STAT-3 inhibition, the GIR in response to peripheral HNGF6A was similar to controls (Fig. 6A). Furthermore, the effect on HGP was completely blocked by central STAT-3 inhibition (Fig. 6B) while effects on skeletal muscle were attenuated (Fig. 6C). This suggests that the effect of peripherally administered HN analog on hepatic insulin action is through the hypothalamus, while the effects on skeletal muscle are due to both central and direct actions.

### No direct effect of HN on hepatic insulin action in vitro

We further probed a possible direct effect of HN on the liver by incubating rat hepatocyte explant cultures with and without HN *in vitro*. When HN was added to the media, there was no effect on glucose production rates (Fig. 6D). We then tested the effects of HN on primary hepatocytes including its ability to induce phosphorylation of STAT-3, as hepatic STAT-3 activation has been linked to insulin action [29]. HN and F6AHN did not elicit significant changes in pAKT^S473^ or pSTAT-3^Tyr705^ in hepatocytes (data not shown), compatible with the lack of effect we demonstrated on glucose production in hepatocytes.

### HNGF6A significantly lowers blood glucose in Zucker diabetic fatty (ZDF) rats

**Figure 7:**
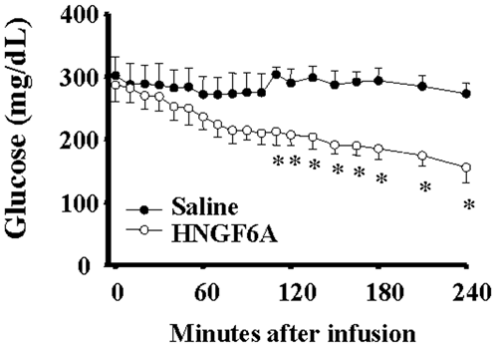
Effect of a single IV dose of HNGF6A on blood glucose levels in chronically catheterized, unstressed Zucker diabetic fatty (ZDF) rats over 4 hours. *Significantly different than saline controls, *P*<0.05.

ZDF rats with a mean glucose level of 293.1±18.7 mg/dL received a single intravenous injection of 100 ug HNGF6A bolus (0.5 ug/uL) (*n* = 5) or 200 uL saline (*n* = 4). As shown in Fig. 7, HNGF6A significantly lowered glucose levels in this diabetic model with effects seen by approximately 90 min and persisting for the remaining 4 hours of blood sampling (*P*<0.05). Indeed, glucose levels decreased by nearly 50% in ZDF rats treated with HNGF6A but were unaltered in rats that received saline.

### Age associated changes in levels of HN in tissues

**Figure 8:**
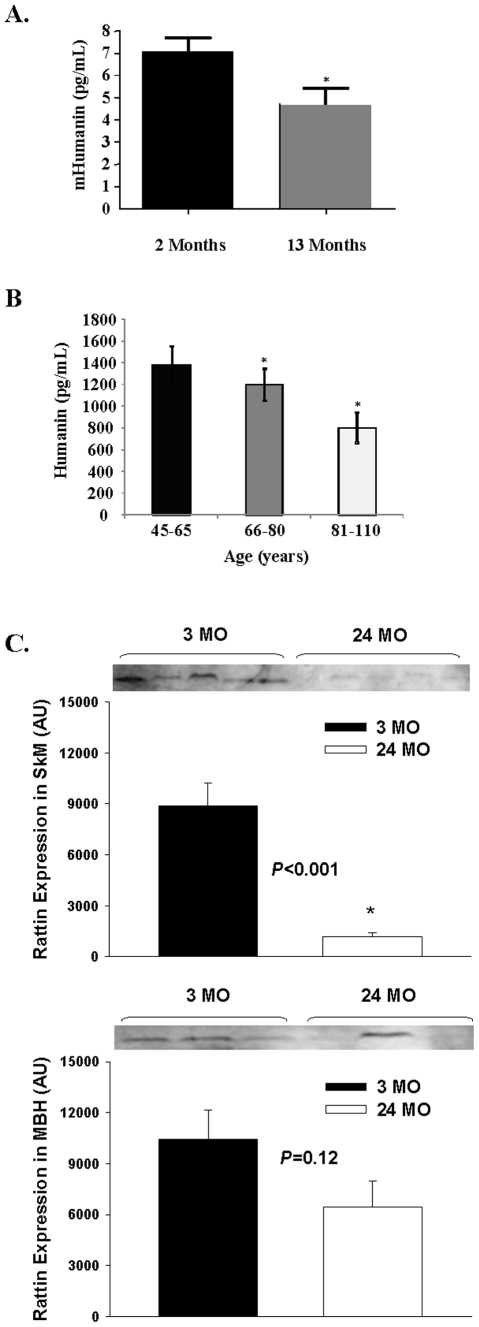
A decline in both plasma and tissue HN levels with aging is observed in rodents and humans. A) HN levels as assessed by ELISA, in young and old mice and B) across age in humans. C) Expression of RN in the hypothalamus and skeletal muscle of young and old rats. *Significantly different from other experimental groups, *P*<0.05.

HN levels in plasma were measured in young and old mice and across age in humans. HN levels decreased with age in both mice (Fig. 8A, *P*<0.05) and humans (Fig. 8B, *P*<0.05). In addition, the expression of the HN homolog RN tended to be decreased with age in the hypothalamus (*P* = 0.12) and was significantly lower in skeletal muscle from old rats (Fig. 8C, *P*<0.05).

## Discussion

We demonstrate here that infusion of HN into the third ventricle of rats, and peripheral infusion of the potent HN analog HNGF6A, improves both hepatic and peripheral insulin sensitivity. We show that, similar to effects on apoptosis, dimerization of HN is essential for its actions on glucose metabolism. We also demonstrate that hypothalamic STAT-3 activation is required for the insulin-sensitizing action of HN. Furthermore, we show that the effects of HN on glucose metabolism are tempered by binding of HN to IGFBP-3 in the hypothalamus, as central infusion of a non-IGFBP-3 binding analog, F6AHN, resulted in a more potent effect on insulin action than HN. We also show that the effect of peripherally administered HN analog on the liver is entirely through the hypothalamus, while the effect on the skeletal muscle may involve both direct and central effects.

The critical role of the MBH in mediating hepatic glucose metabolism has been unraveled by prior studies with leptin, insulin and IGF-1. In the studies presented here, the hypothalamus appears to play a key role in mediating the effects on hepatic insulin sensitivity in response to the administration of both central and peripheral HN and its analogs. Remarkably, centrally-administered HN dramatically increased GIR not only due to effects on HGP, but also through an increase in peripheral glucose uptake, demonstrating that HN is the first centrally-acting peptide to favorably modulate glucose uptake in skeletal muscle. The autonomic nervous system may serve as the efferent in mediating the effects of HN on peripheral glucose metabolism. Hypothalamic nuclei have been shown to influence HGP through the vagus nerve [30], while leptin and IGF-1 have been shown to effect output through the sympathetic nervous system [31], [32]. More studies are needed to specifically evaluate the role of autonomic nervous system in mediating the effects of HN.

This study also provides evidence that HN and IGFBP-3 not only have opposing roles on apoptosis but also on peripheral insulin action via the hypothalamus. The opposing roles of these two peptides on glucose homeostasis and apoptosis are especially interesting when one considers the close relationship between insulin resistance and cell survival in AD. In fact, defects in glucose utilization, a striking reduction in insulin receptor mRNA levels, and attenuated insulin signaling, as evidenced by decreased IRS-1, PI3K, and pAKT have been demonstrated in AD [33]. On the other hand, insulin sensitizers such as thiazolinediones improve cognitive function in mouse models as well as humans with early AD, highlighting the role of insulin resistance in this condition [34]. While IGFBP-3 production is increased in AD brain [35], HN is expressed in the non-apoptotic hippocampal regions of Alzheimer's patients' brains and is absent in senile plaques [3]. Taken together, the improvement in insulin sensitivity demonstrated with central HN may be one of the primary mechanisms by which HN regulates cell survival and, therefore, may provide an additional potential mechanism by which HN is protective against AD.

Molecular manipulations of HN at key amino acids have been shown to offer significant increases in its potency or chemical characteristics. For instance, a change at position 14, [Gly^14^]-HN (S14G, HNG), has been shown to enhance neurosurvival activity and also offers added protection against memory impairment [36] and stroke [37]. A substitution of phenylalanine in the 6^th^ position with alanine (F6A, F6AHN) alters the binding of HN to IGFBP-3 and we show here that this substitution significantly enhances its central effect on glucose metabolism. Interestingly a novel HN analog termed HNGF6A, which we created by combining both changes at position 6 and 14, dramatically increases its ability to modulate insulin action and offers a mode of administration that is more clinically applicable. We also show here that HNGF6A not only improves insulin sensitivity during a clamp, but also significantly improves blood glucose levels in ZDF rats with a single dose. The potential of HNGF6A as a treatment for T2DM is an exciting observation which warrants further investigation, including chronic treatment of diabetic models with this peptide.

Another important observation from this study is that intact STAT-3 signaling in the hypothalamus is necessary for the effects of HN on glucose metabolism. This is in partial agreement with prior studies which have pinpointed the mechanism by which HN confers neuronal protection to the activation of tyrosine kinases and STAT-3 phosphorylation [14]. STAT-3 phosphorylation and activation in the hypothalamus is well known to play a pivotal role in the regulation of energy homeostasis. For example, neuronal STAT-3 KO mice are morbidly obese, hyperphagic and diabetic [38]. This effect is due at least in part to attenuated leptin signaling in the hypothalamus. This has been directly demonstrated in rats by co-infusion of a STAT-3 inhibitor with leptin which resulted in attenuation of the actions of leptin on food intake and glucose metabolism [23]. Likewise, we show that co-infusion of a STAT-3 inhibitor with HN into the third ventricle completely attenuates the ability of HN to modulate peripheral insulin action. Indeed, when STAT-3 signaling was inhibited in the hypothalamus, the effects of peripherally administered HN analog on liver was completely abolished, once again emphasizing the role of the hypothalamus in mediating the hepatic effects of HN and its analogues. Furthermore, the absence of any effect on phosphorylation levels of other signaling kinases, including AKT, demonstrates that the effects of HN are independent of hypo thalamic insulin signaling.

The potential of anti-apoptotic therapy for neurodegeneration has been gaining attention [39], and growth factors of various types including IGF-1 and other neuro-survival peptides [39], [40] have been considered. While the role of HN in neuroprotection has been substantially characterized and HN has been proposed to have enormous therapeutic potential for neurodegenerative diseases, the studies described here comprise the first evidence demonstrating a role for HN in glucose metabolism. Considering that diseases associated with aging such as T2DM and AD have been proposed to be associated with mitochondrial dysfunction [41], [42], it is especially interesting that the mitochondrial-derived peptide HN modulates them. In summary, these data demonstrate for the first time that HN regulates peripheral insulin action. The link between the decrease in circulating levels of HN as well as levels in hypothalamus and skeletal muscle with age, in parallel with the increase in age- associated diseases such as AD and T2DM is extremely intriguing and warrants further investigation. HN or its non-IGFBP-3 binding analogues may provide potential therapeutic options for prevention or treatment of at least two age-related diseases, namely impaired carbohydrate metabolism/T2DM and neurodegeneration.
